# *Mycoplasma hominis* bloodstream infection and persistent pneumonia in a neurosurgery patient: a case report

**DOI:** 10.1186/s12879-022-07137-4

**Published:** 2022-02-21

**Authors:** Qiang Wang, Xiaofang Tang, Stijn van der Veen

**Affiliations:** 1 Department of Clinical Laboratory, The Second Affiliated Hospital of Zhejiang Chinese Medical University, Hangzhou, 310005 People’s Republic of China; 2 Cadre Health Care Division, Zhejiang Hospital, Hangzhou, 310013 People’s Republic of China; 3 Department of Microbiology, and Department of Dermatology of Sir Run Run Shaw Hospital, School of Medicine, Zhejiang University, 866 Yuhangtang Road, Hangzhou, 310058 People’s Republic of China; 4 State Key Laboratory for Diagnosis and Treatment of Infectious Diseases, Collaborative Innovation Center for Diagnosis and Treatment of Infectious Diseases, The First Affiliated Hospital, School of Medicine, Zhejiang University, Hangzhou, People’s Republic of China

**Keywords:** Bloodstream infection, Cerebral hemorrhage, Prostate abscess, *Mycoplasma hominis*, Pneumonia, Case report

## Abstract

**Background:**

*Mycoplasma hominis* is typically associated with a urogenital tract infection, while its association with bacteremia and pneumonia is rare and therefore easily overlooked. Here we report a *M. hominis* bloodstream infection and pneumonia in a surgical patient.

**Case presentation:**

A 56-year-old male with symptoms of pneumonia underwent microsurgery and decompressive craniectomy after a left basal ganglia hemorrhage. The patient recovered well from surgery, but pulmonary symptoms progressively worsened, with antimicrobial therapies seemingly ineffective. Culturing of bilateral blood samples resulted in pin-point-sized colonies on blood agar plates, which were subsequently identified as *M. hominis* by matrix-assisted laser desorption/ionization time-of-flight mass spectrometry. Furthermore, sequencing of bronchoalveolar lavage samples also identified *M. hominis* as the main pathogen responsible for the pulmonary symptoms. The *M. hominis* strain was ciprofloxacin resistant, but susceptible to doxycycline and moxifloxacin. Doxycycline and moxifloxacin were subsequently used in a successful combination therapy that finally alleviated the patient’s fever and resulted in absorption of pleural effusion. At 1-month follow-up, following complaints of dysuria, a prostate abscess containing *M. hominis* was detected as the likely primary source of infection. The abscess was successfully drained and treated with doxycycline.

**Conclusions:**

*Mycoplasma hominis* should be considered as a source of bloodstream infections and pneumonia, particularly when the response to standard antimicrobial therapy is limited. In this case, effective antimicrobial therapy was only commenced after identification of *M. hominis* and antimicrobial susceptibility testing.

## Background

*Mycoplasma hominis* is commonly found in the female and male genital tract and is associated with a variety of urogenital diseases [1]. However, *M. hominis* is rarely detected in bloodstream infections or isolated from blood cultures [2]. Here we report a case of a postoperative *M. hominis* bloodstream infection after neurosurgical intervention on an acute cerebral hemorrhage in a patient displaying pneumonia. This bacterium was isolated from anaerobic blood cultures and furthermore identified by sequencing of bronchoalveolar lavage (BAL) samples after progressive worsening of pulmonary symptoms. Only after correct identification of *M. hominis*, effective therapy was commenced for this difficult to diagnose infection. At 1-month follow-up, a *M. hominis*-containing prostate abscess was detected as the likely primary infection site, which was subsequently successfully treated.

## Case presentation

**Fig. 1:**
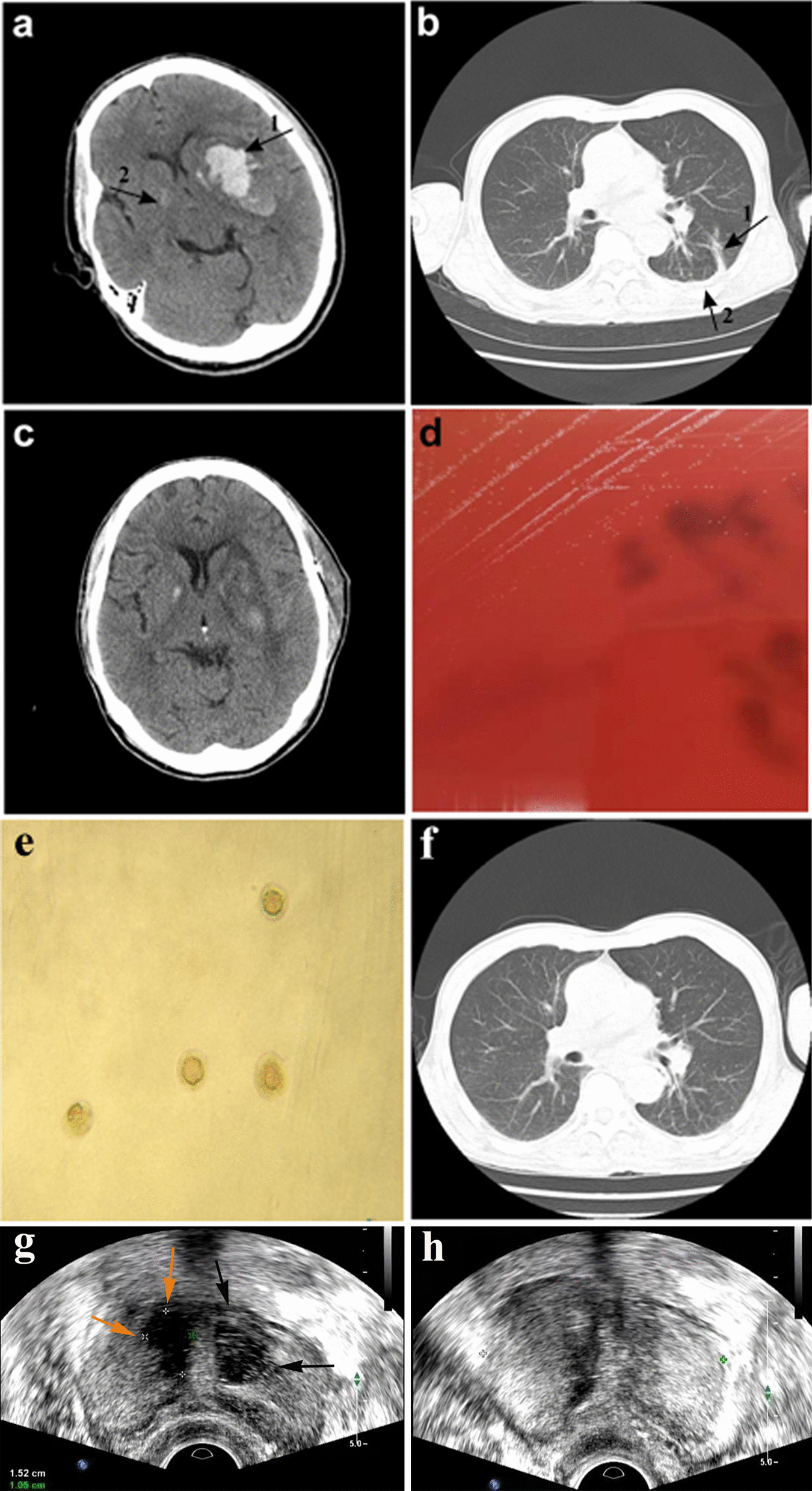
Computed tomography (CT) images of the brain and chest before and after microsurgery or antimicrobial treatment. **a** Brain CT scan of the patient showing cerebral hemorrhage on day 1 of hospital admission. Arrow 1: left basal ganglia hematoma. Arrow 2: right basal calcification. **b** Chest CT scan of the patient on day 8 after hospital admission showing fluid accumulation in the lungs. Arrow 1: inflammatory lesions in the lower lobe of the left lung. Arrow 2: small amount of pleural effusion on both sides of the chest. **c** CT scan of the brain on day 16 after hospital admission showing good recovery after surgery. **d** Development of pin-point-sized colonies after 48 h sub-culturing of the left side anaerobic culture bottle on Columbia blood agar at 37 °C in the presence of 5% CO_2_. **e** Development of typical *M. hominis* fried-egg-shaped colonies after sub-culturing on A7 agar. **f** Chest CT scan of the patient on day 33 after hospital admission showing absorption of chest effusion. **g** Transrectal ultrasound (TRUS) scan of the prostate at 1-month follow-up showing right-side (yellow arrow) and left-side (black arrows) prostate abscesses. **h** TRUS scan of the prostate 15 days after the drainage procedure showing return to a healthy state

July 2019, a 56-year-old male was admitted to the Emergency Department of the Second Affiliated Hospital of Zhejiang Chinese Medical University, Hangzhou, China, after suffering from unconsciousness for five hours. The patient displayed inability to stand, limb twitching, nausea and vomiting, and he did not have an obvious inducement for sudden unconsciousness. Physical examination upon admission showed that he was able to open his eyes upon pain stimulation, and he furthermore showed aphasia, bilateral pupils with a diameter of 3 mm, sensitivity to light, right limb muscle strength Grade II, bilateral breathing sounds and rales, and right Pap sign (+) (indicating left cerebral infarction). A cerebral computed tomography (CT) scan identified a left basal ganglia hematoma and right basal calcification (Fig. 1a). Furthermore, the patient showed pulmonary emphysema with inflammation of the lower lobe of the left lung and a small amount of pleural effusion on both sides. The patient’s family reported that before this episode the patient was in good health and did not suffer from diabetes or any immune diseases and that he was not on any medications. Furthermore, no problems were detected by transesophageal echocardiography and the patient did not display any symptoms of endocarditis, indicating that a mycotic aneurisms was unlikely. After emergency treatment to control blood pressure, lowering intracranial pressure and hemostasis, re-examination by cranial CT scan showed that bleeding had progressed. The patient was then referred to the Department of Neurosurgery and on the same day he underwent microsurgery and decompressive craniectomy. Approximately 30 ml of the hematoma was removed during the surgery, and a drainage tube was placed in the hematoma cavity after hemostasis. The patient was returned to the ward with a body temperature of 36.8 °C and tracheal intubation because of preoperative vomiting and lung inflammation and he was treated empirically with piperacillin-tazobactam (4.5 g IV q8h). The drainage tube from the hematoma cavity was removed on the 3rd day, while a pre-operatively placed catheter was removed on the 7th day after admission and both remained negative in subsequent culture tests.

On the 8th day after hospital admission, the patient had a sudden fever with a body temperature of 38.6 °C and a lung CT scan showed inflammatory lesions in the lower lobe of the left lung, and a small amount of pleural effusion on both sides of the chest (Fig. 1b). Due to empirical therapy considerations for a likely Gram-negative bacterial infection, piperacillin-tazobactam was discontinued and meropenem therapy (0.5 g IV q8h) was initiated. The patient’s pre-operatively placed urinary catheter was removed on the 15th day after hospital admission and both the catheter and urine samples remained culture negative, while urine routine was normal. However, the patient’s body temperature remained elevated and on the 16th day after hospital admission, the patient started to cough-up yellow sticky sputum. However, he did not display chest tightness or shortness of breath and his right limb hemiplegia and scalp incision healed well (Fig. 1c). On the 18th day after admission, his white blood cell (WBC) counts were 9.6 × 10^9^/L with a neutrophil % of 86.5% and C-reactive protein (CRP) levels were 17.2 mg/L. Therefore, bilateral blood samples were drawn for aerobic (BacT/ALERT FA, bioMérieux) and anaerobic (BacT/ALERT SN, bioMérieux) culturing to investigate a possible bacterial infection. In addition, antimicrobial treatment was modified to meropenem (0.5 g IV q8h) in combination with ceftriaxone (2 g IV qd). On the 21st day after admission, one of the anaerobic culture bottle was positive for growth, but no bacteria were identified by Gram-staining. Subsequent sub-culturing resulted in pin-point-sized colonies on Columbia blood agar plates (bioMérieux) by day 23 after admission (Fig. 1d), which were identified as *M. hominis* by matrix-assisted laser desorption/ionization time-of-flight mass spectrometry (MALDI-TOF MS; VITEK MS, bioMérieux) and further confirmed by16S rRNA sequencing and growth as typical fried-egg-shaped colonies on A7 agar (Fig. 1e). In the meantime, treatment was modified on the 22nd day after admission to linezolid (600 mg IV q12h) combined with meropenem (0.5 g IV q8h) and discontinued for ceftriaxone. Furthermore, a lumbar puncture on the 22nd day did not display any evidence of cerebrospinal fluid (CSF) infection by routine laboratory tests. A BAL was also performed on the 22nd day given the persistence of pulmonary symptoms. BAL fluid was used for next-generation sequencing (BGI-Nanjing) and bioinformatics analysis as described previously [3]. Shortly, DNA was extracted using the TIANamp Micro DNA Kit (DP316, TIANGEN BIOTECH) and generated DNA Nanoballs (DNB) were sequenced by the MGISEQ-2000 platform, followed by pathogen identification after alignment with genome sequences obtained from the Pathogens Metagenomics Database (PMDB). Sequencing results on the 24th day identified *M. hominis* as the main pathogenic bacterium in the BAL fluid (Table 1). After identification of *M. hominis* on day 23 after hospital admission, treatment was modified to ciprofloxacin (400 mg IV q12). However, by day 25 after admission antimicrobial susceptibility testing according to the broth microdilution method [4] showed that the *M. hominis* strain was resistant to ciprofloxacin and susceptible to doxycycline and moxifloxacin. Therefore, treatment was modified to doxycycline (100 mg po bid) combined with moxifloxacin (400 mg IV qd). The following 8 days, the patient’s fever gradually declined and his lung inflammation improved with clear absorption of chest effusion (Fig. 1f). On the 33th day after admission, based on requests by the patient’s family the patient was transferred to a hospital in the patient’s hometown and continued for another 7 days on doxycycline (100 mg po bid) combined with moxifloxacin (400 mg IV qd).

**Table 1:** Next generation sequencing results of the bronchoalveolar lavage (BAL) sample

Genus	Number of sequence reads	Species	Number of sequence reads
*Streptococcus*	78	*Streptococcus mitis* ^a^	27
		*Streptococcus agalactiae* ^a^	4
		*Streptococcus constellatus*	7
*Haemophilus*	14	*Haemophilus parainfluenzae*	8
*Enterococcus*	5	*Enterococcus faecium*	5
*Mycoplasma*	9996	*Mycoplasma hominis*	9697

^a^Normal flora present in the oral samples

At 1-month follow-up, cerebral CT examination displayed no abnormalities, while chest CT revealed showed obvious absorption of the pulmonary inflammation. Furthermore, the patient’s body temperature and WBC counts were normal. However, the patient complained of dysuria and perineal pain and he was therefore referred to the urologist. Rectal examination revealed an enlarged, firm prostate with a middle lateral nodule, but without signs of fluctuations. But urine cultures remained negative. The patient was then referred for transrectal ultrasound (TRUS) with a pre-diagnosis of prostate abscess. TRUS showed and enlarged prostate (50 × 50 × 41 mm) and multiple lesions in the transition zone of the prostate. These thick-walled hypo-echoic lesions with internal echogenicities resembled abscesses. Both the right-side lesion (15 × 11 mm) and the left side lesion (18 × 12 mm) were uniloculated (Fig. 1g). The patient was directly hospitalized and started on intravenous doxycycline (100 mg IV q12), followed 2 days later by ultrasound-guided transperineal drainage of the abscesses and flushing with doxycycline. The aspirate subsequently cultured *M. hominis*, but whole-genome sequencing for comparison with the earlier isolate was not performed. A 24 h follow-up TRUS control was abscess negative, but hypoechoic areas that seemed like inflammatory changes were seen in the abscess location. The patient was discharged from the hospital, as his clinical symptoms were resolved. The patient was maintained on oral doxycycline (100 mg po bid) for 14 days after the intervention. On the follow-up TRUS control 15 days after the procedure, abscess recurrence was not seen (Fig. 1h), while at the 1-month follow-up TRUS lesions had disappeared and the prostate gland was no longer enlarged. Cerebral and chest CT examinations were performed at 1-year follow-up and displayed no abnormalities.

## Discussion and conclusions

*Mycoplasma hominis* is a rare cause of bloodstream infections in the neurosurgical patient population [5, 6]. There have been individual cases reported where *M. hominis* was the causative agent of meningitis after neurosurgery, which was possibly associated with an indwelling tube infection [6]. Although, *M. hominis* brain abscess infections after neurosurgery are rarely observed [7, 8], considering the case report of systemic *M. hominis* infection after craniotomy by an unqualified device [8], it cannot be ruled out that systemic *M. hominis* infection in our case might be through the surgical wound. However, it is unlikely that the cerebral hemorrhage was related with the *M. hominis* infection, since no signs of a CSF infection was identified after lumbar puncture and cultures of the drainage tube from the hematoma cavity remained negative.

*Mycoplasma hominis* is difficult to diagnose due to the delicate culture methods required to yield a positive result [9]. Typical broad-spectrum antibiotics, such as vancomycin, ceftriaxone, meropenem and metronidazole are ineffective against *M. hominis*. In this case, *M. hominis* was identified by anaerobic culturing of blood samples. Pre- and post-operative pulmonary inflammation was evident, but the inflammatory markers WBC and CRP were not strikingly high. Because *M. hominis* was not considered as the causative agent for the patient’s pulmonary symptoms, they progressively aggravated due to ineffective antimicrobial therapy, ultimately resulting in a small amount of effusion on both sides of the chest. After identification of *M. hominis* from blood cultures and BAL sample sequencing and initiation of targeted treatment, absorption of pleural effusion was observed. Blood cultures of *M. hominis* are easily missed, but dissemination of this bacterium from the respiratory tract into the bloodstream is very uncommon [10]. More likely, pneumonia was the consequence of hematogenous dissemination from the *M. hominis*-positive prostate abscess detected at 1-month follow-up.

*Mycoplasma hominis* is a common colonizer of the genitourinary system, where it can cause genitourinary tract infections [1, 11]. Under normal circumstances, it is unable to penetrate the submucosal layers of the genitourinary tract. The most common cause of *M. hominis* infection outside of the genitourinary system is through incisions after gynecological and obstetrical interventions, or after placement of a catheter [9]. In our case, both the urinary catheter removed 1 week after onset of fever and urine samples remained culture negative. Furthermore, the patient did not display any symptoms of dysuria at that stage and so the urogenital system was not suspected as a possible origin of the infection. Only during the 1-month follow-up, the patient complaint of dysuria, which was caused by a prostate abscess containing *M. hominis*. Prostate abscesses are most commonly encountered in patients with a compromised immune system or diabetes and frequently the result of bacterial prostatitis, although hematogenous dissemination from distant primary infection sites have also been recorded [12]. However, given that *M. hominis* as a slow growing organism generally has an extended subclinical incubation period, the developing prostate abscess was likely the primary infection site in this patient.

In conclusion, *M. hominis* is not a commonly suspected cause for bloodstream infections or pneumonia and identification is challenging due to the requirement for prolonged anaerobic culturing. In this case, positive identification of *M. hominis* as the source of infection and subsequent antimicrobial susceptibility testing were a prerequisite for initiation of effective antimicrobial therapy.

## Data Availability

Not applicable.

## Abbreviations

BAL: Bronchoalveolar lavage
CT: Computed tomography
IV: Intravenous
CRP: C-reactive protein
MALDI-TOF MS: Matrix-assisted laser desorption/ionization time-of-flight mass spectrometry
CSF: Cerebrospinal fluid
WBC: White blood cell
